# Novel JAK Inhibitors to Reduce Graft-Versus-Host Disease after Allogeneic Hematopoietic Cell Transplantation in a Preclinical Mouse Model

**DOI:** 10.3390/molecules29081801

**Published:** 2024-04-16

**Authors:** Sena Kim, Peter Ruminski, Megh Singh, Karl Staser, Kidist Ashami, Julie Ritchey, Sora Lim, John F. DiPersio, Jaebok Choi

**Affiliations:** Division of Oncology, Department of Medicine, Washington University School of Medicine, St. Louis, MO 63110, USA; senakim@wustl.edu (S.K.); pgruminski@wustl.edu (P.R.); meghsingh@wustl.edu (M.S.); kwstaser@gmail.com (K.S.); kashami@g.harvard.edu (K.A.); jritchey@wustl.edu (J.R.); slim22@wustl.edu (S.L.); jdipersi@wustl.edu (J.F.D.)

**Keywords:** graft-versus-host disease, interferon-gamma receptor, Janus kinases, Janus kinase inhibitor

## Abstract

Allogeneic hematopoietic cell transplantation (allo-HCT) is a highly effective, well-established treatment for patients with various hematologic malignancies and non-malignant diseases. The therapeutic benefits of allo-HCT are mediated by alloreactive T cells in donor grafts. However, there is a significant risk of graft-versus-host disease (GvHD), in which the donor T cells recognize recipient cells as foreign and attack healthy organs in addition to malignancies. We previously demonstrated that targeting JAK1/JAK2, mediators of interferon-gamma receptor (IFNGR) and IL-6 receptor signaling, in donor T cells using baricitinib and ruxolitinib results in a significant reduction in GvHD after allo-HCT. Furthermore, we showed that balanced inhibition of JAK1/JAK2 while sparing JAK3 is important for the optimal prevention of GvHD. Thus, we have generated novel JAK1/JAK2 inhibitors, termed WU derivatives, by modifying baricitinib. Our results show that WU derivatives have the potential to mitigate GvHD by upregulating regulatory T cells and immune reconstitution while reducing the frequencies of antigen-presenting cells (APCs) and CD80 expression on these APCs in our preclinical mouse model of allo-HCT. In addition, WU derivatives effectively downregulated CXCR3 and T-bet in primary murine T cells. In summary, we have generated novel JAK inhibitors that could serve as alternatives to baricitinib or ruxolitinib.

## 1. Introduction

Allogeneic hematopoietic cell transplantation (allo-HCT) is an effective immunotherapy for patients with both malignant disorders and nonmalignant disorders, including bone marrow failure states, hemoglobinopathies, and thalassemia [1,2]. The major therapeutic benefit of allo-HCT primarily relies on the graft-versus-leukemia (GvL) effect mediated by mature T cells present in the donor hematopoietic cell graft [3]. However, graft-versus-host disease (GvHD) is a significant challenge after allo-HCT, as transplanted donor T cells recognize and damage not only malignant cells, but also multiple host tissues and organs, as foreign [4]. Despite prophylactic treatments with immune-suppressive agents, up to 30–60% of transplantation recipients still develop GvHD [5]. Thus, GvHD remains a significant barrier to the success of allo-HCT, impacting overall quality of life.

Janus kinase (JAK) is a family of cytoplasmic non-receptor tyrosine protein kinases that play an important role in cytokine and growth factor signaling through signal transducers and activators of transcription (STATs) [6]. JAK-STAT signaling regulates the transcription of genes associated with the pathogenesis of inflammatory and autoimmune diseases including rheumatoid arthritis, psoriasis, and inflammatory bowel disease. The JAK family comprises four members (JAK1, JAK2, JAK3, and TYK2) found in all vertebrates, and JAK inhibitors approved for clinical use exhibit a broad range of inhibition profiles for JAKs [7,8]. Ruxolitinib (RUX) was the first FDA-approved JAK inhibitor in 2011, as a selective JAK1/JAK2 inhibitor with moderate affinity against JAK3 and TYK2. Subsequently, additional JAK inhibitors, including tofacitinib (a pan JAK inhibitor with greater selectivity for JAK1/JAK3), baricitinib (BARI, a JAK1/JAK2), upadacitinib (JAK1), filgotinib (JAK1), pacritinib (JAK2), and abrocitinib (JAK1), have been approved for treating various inflammatory and autoimmune diseases, such as atopic dermatitis, psoriasis, alopecia areata, psoriatic arthritis, juvenile idiopathic arthritis, rheumatoid arthritis, ankylosing spondylitis, ulcerative colitis, and most recently, vitiligo [9,10,11]. 

We were the first to demonstrate that pharmacologic inhibition of JAK1 and JAK2 using BARI or RUX effectively prevents and treats GvHD in our preclinical mouse models of allo-HCT [12,13]. We also previously identified interferon gamma receptors (IFNGRs) and interleukin-6 receptors (IL-6Rs) as optimal targets for the complete prevention of GvHD [13]. Given that both IFNGR and IL-6R signaling are mediated by both JAK1 and JAK2, we additionally confirmed that the balanced inhibition of JAK1/JAK2 is important to block both IFNGR and IL-6R signaling for optimal GvHD prevention [13,14]. While both BARI and RUX show well-balanced inhibitory activity against JAK1/JAK2 to effectively block IFNGR and IL-6R signaling, we found that BARI is superior to RUX in preventing GvHD by increasing regulatory T cells (Tregs) through sparing JAK3 signaling in our preclinical mouse models of allo-HCT [13,15]. This may be partially due to the better pharmacokinetic (PK) profile of BARI compared to RUX. BARI has a reported plasma protein binding of 50% in humans and a terminal plasma half-life of ~8.5 h in humans, with very little metabolism and elimination mostly as parent, whereas RUX has a reported plasma protein binding of 97%, a terminal plasma half-life of ~2 h, and extensive metabolism (>99%) mostly in the liver via CYP(3A4) [16,17]. In our current study, this pharmacokinetic profile, indicating BARI’s superiority over RUX, is similarly observed in mice. 

The US Food and Drug Administration (FDA) approved RUX (aka Jakavi^®^) for the treatment of steroid-refractory acute and chronic GvHD in 2019 and 2021, respectively, while BARI is still pending approval. However, RUX and BARI may elevate the risk of serious and potentially life-threatening adverse events, including pulmonary embolism and arterial and/or deep venous thrombosis [18]. 

In this study, we have designed and synthesized BARI analogues named WU derivatives to generate novel JAK1/JAK2 inhibitors which might be safer and have improved selectivity profiles and PK properties compared to RUX and BARI. The WU derivatives reported here have potent inhibitory activity against JAK1 and JAK2, with some sparing JAK3 and TYK2 to varying degrees. Subsequently, we investigated the effects of these WU derivatives on murine GvHD, demonstrating a potential for the WU derivatives to improve overall survival with less GvHD in our preclinical model of allo-HCT. Our data suggest that WU derivatives could serve as promising alternatives to RUX and BARI. 

## 2. Results

### 2.1. Design and Synthesis of Novel JAK1/JAK2 Inhibitors: WU Derivatives

**Figure 1:**
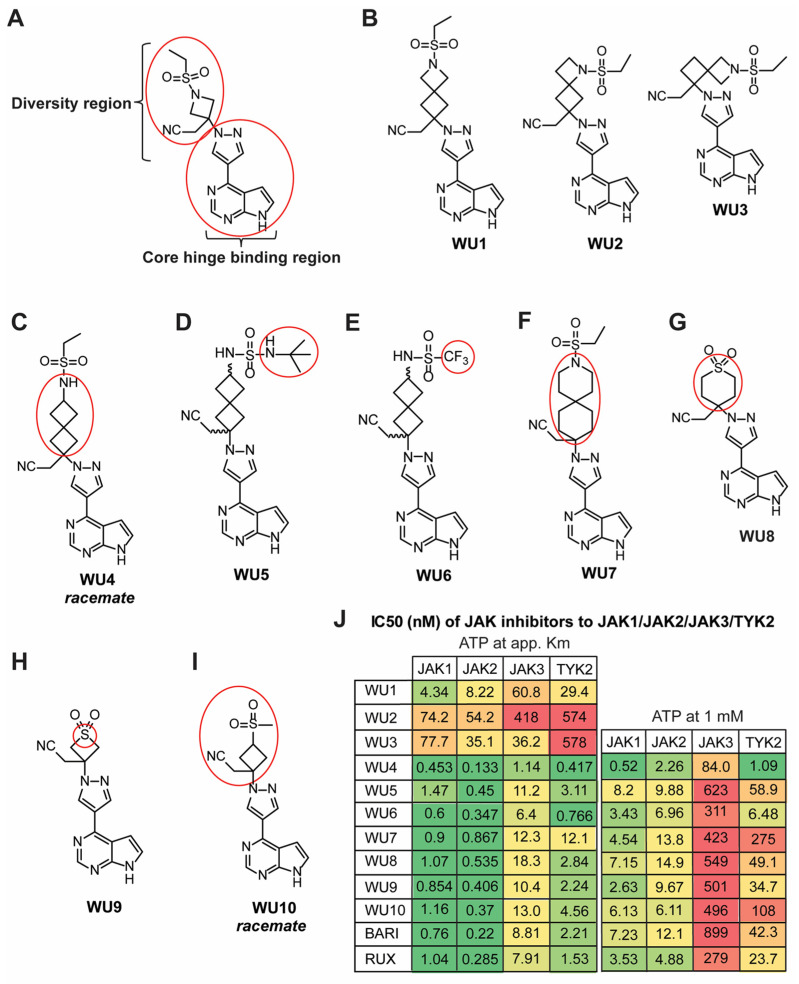
Chemical structures of BARI and novel BARI analogues. (**A**) Structure of BARI, new analogues designed to modify the diversity region of BARI attached to the preserved quaternary carbon-linked cyanomethyl group (red circle in diversity region) while retaining the pyrrolopyrimidine pyrazole core (red circle in the hinge binding region). (**B**–**I**) Structures of generated BARI analogues (WU derivatives). Red circles indicate modified functional groups of the selected analogues in the diversity region of BARI. (**J**) Inhibitory activity of WU derivatives, BARI, and RUX against JAKs was tested in the Z’-LYTE enzyme assay.

Given that BARI showed superior capacity in improving GvHD with prolonged efficacy in our preclinical mouse model of allo-HCT compared to RUX [12,13], we developed a focused and targeted effort to design and synthesize novel JAK1/JAK2-selective analogues based upon structures and structure–activity relationships of currently known JAK inhibitors, particularly BARI (Supplementary Information). We retained the hinge-binding pyrrolopyrimidine pyrazole core of the BARI structure as well as keeping the cyanomethyl quaternary carbon moiety of the diversity region constant (Figure 1A). We directed our analogue targets on modifications to the rest of the diversity region attached to the quaternary carbon. One such targeted scaffold, an azaspiro functionality, can be an efficient surrogate for the piperidine, piperazine, and azetidine groups (all of which are core functionalities of many JAK inhibitors), and may provide improved solubility and stability properties in addition to an even better JAK1/JAK2 selectivity profile. To date, azaspiro JAK inhibitors have not been reported, and thus, we aimed to design and optimize a novel class of JAK analogues for the prevention and treatment of GvHD in this study.

WU1, which extends the four-membered azetidine of BARI via fusion with another four-membered ring, forming the azaspiro[3.3] heptane analogue, while keeping the ethyl sulfonamide moiety intact, attenuates inhibition across all four JAK family members compared to BARI (Figure 1B, left panel). The loss of inhibitory activity against JAKs is also observed in the other azaspiro[3.3] heptane isomers, WU2 and WU3 (Figure 1B, middle and right panels). Interestingly, however, when the nitrogen of WU1 in the four-membered ring is moved external to the spirocycle, forming the 2-ethyl spiro[3.3] heptane sulfonamide WU4, inhibitory activity is significantly increased against all JAKs (Figure 1C). Analogues of WU4, the N-t-butyl sulfamide WU5 (Figure 1D), and the trifluoromethyl sulfonamide WU6 (Figure 1E) are slightly less potent than WU4 against JAK1/JAK2, but are somewhat more selective against JAK3. In another interesting twist, when the nitrogen of the ethyl sulfonamide moiety is moved back into a six-membered spirocycle, the azaspiro[5.5] undecane WU7, it provides the most balanced JAK1/JAK2 inhibition while sparing JAK3/TYK2 evenly (Figure 1F). In addition to the spirocycle analogues described above, the six-membered 1,1-dioxo-tetrahydro-thiopyran analogue WU8 provides for increased selectivity over JAK3 compared to WU4 (Figure 1G), as does its 4-membered analogue, thietane-1,1-dioxide WU9 (Figure 1H). Lastly, replacing the azetidine nitrogen of BARI with a carbon, the methane sulfonyl cyclobutane WU10 (Figure 1I), does not alter the JAK inhibitory profile to any significant degree, indicating that this nitrogen is not critical for JAK inhibition per se.

The structures of the aforementioned analogues are depicted in Figure 1B–I. The half-maximal inhibitory concentration (IC_50_) of WU derivatives to JAK1, JAK2, JAK3, and TYK2 was determined in cell-free isolated JAK enzyme assays utilizing SelectScreen Kinase profiling (Figure 1J). When comparing IC_50_ values between the ATP concentration at app. Km and 1 mM, smaller differences can represent a stronger affinity of the drugs to the kinase, whereas greater differences can indicate a weaker affinity of the drug to the kinase. Among them, BARI exhibited the weakest affinity (high ratio of drug concentration from ATP at app. Km to 1 mM) against JAK1, JAK2, and JAK3, despite effectively inhibiting JAK1/JAK2 (Figure 1J).

**Figure 2:**
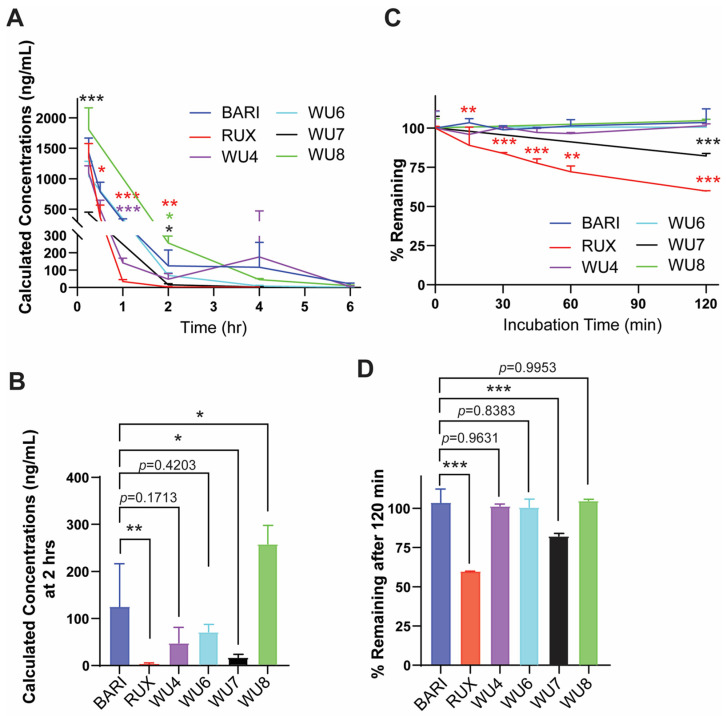
PK and HLM data of BARI, RUX, and the WU derivatives. (**A**,**B**) Mouse PK following subcutaneous administration of WU derivatives (2 mg/kg) to CD-1 mice. The concentration of WU derivatives, BARI, and RUX in mouse plasma was measured at multiple time points. (**C**,**D**) The graph shows intrinsic clearance values (% remaining of drugs) of WU derivatives, BARI, and RUX in incubations with human liver microsomes. * *p* < 0.05, ** *p* < 0.01, and *** *p* < 0.001. The colors of the asterisks indicate statistical significance in panels (**A**,**C**); red, purple, black, and green indicate RUX, WU4, WU7, and WU8 compared to BARI, respectively. All error bars are represented as mean ± standard deviation.

Next, we examined mouse PKs of some of the WU derivatives (WU4, WU6-8) to characterize their plasma concentrations following subcutaneous (s.c.) injection compared to BARI and RUX, as well as an in vitro metabolism profile in a human liver microsome (HLM) assay. The mouse PK study of the WU derivatives, BARI, and RUX quantified the concentration of each drug in plasma at several time points up to 6 h after s.c. injection (2 mg/kg) to CD-1 mice. BARI exhibited superior PK compared to RUX, maintaining significantly higher plasma drug levels for up to 2 h after injection (Figure 2A), which has similarly been observed in humans [16,17]. In comparison to BARI, WU8 showed a significant increase in plasma drug concentration at 2 h post-dose, but with BARI maintaining higher plasma concentrations at 4 h (Figure 2A). However, WU7 showed a significant reduction in plasma concentration at 2 h after injection (Figure 2B). WU4 had comparable PK to BARI throughout all time points (Figure 2A,B). Subsequently, we assessed the in vitro metabolic stability and clearance values of WU derivatives in an HLM assay. All of the tested WU derivatives, except for WU7, showed excellent metabolic stability comparable to BARI (Figure 2C,D). The diminished HLM metabolic stability profile exhibited by WU7 corresponded to the poor plasma PK profile demonstrated in the mouse PK studies (Figure 2B). All these pharmacokinetic results suggest that our WU derivatives demonstrated improved or comparable properties compared to BARI, which was significantly better than RUX (Figure 2).

### 2.2. Inhibitory Effect of WU Derivatives on the Chemokine Receptor CXCR3 and STAT Phosphorylation in Primary T Cells

**Figure 3:**
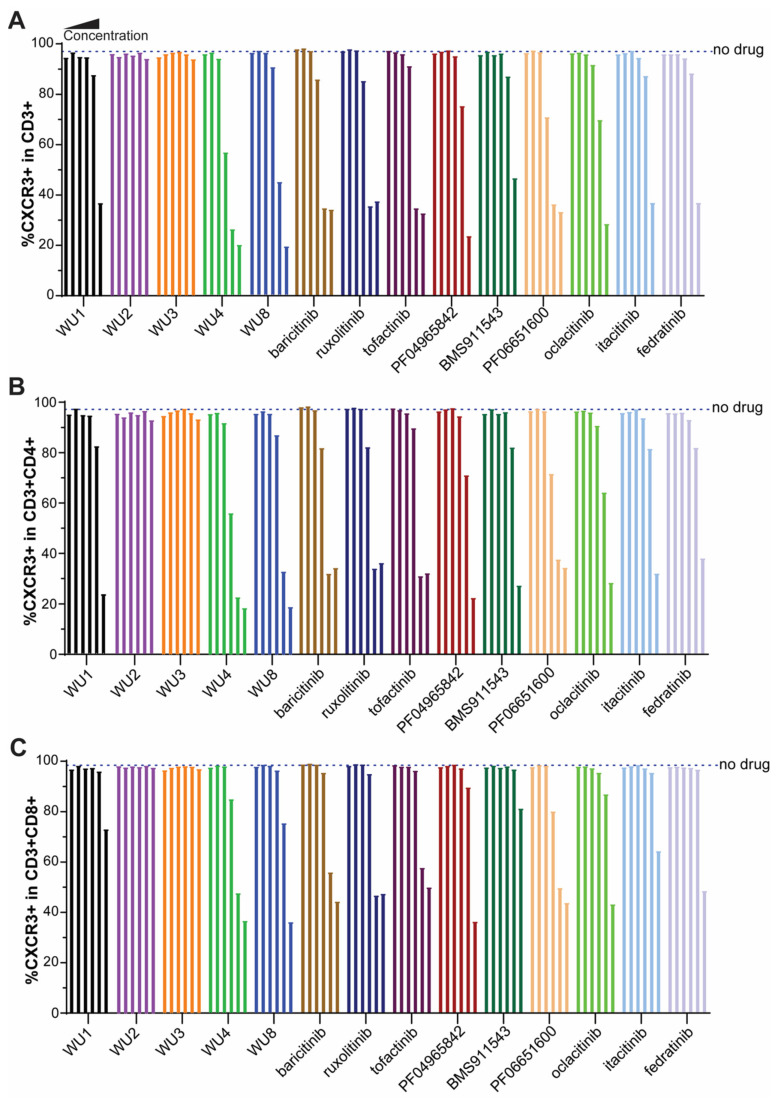
Inhibitory effect of WU derivatives on the expression of CXCR3 compared to commercially available JAK inhibitors in primary murine T cells. Primary murine T cells were isolated from B6 mice and treated with WU derivatives or known JAK inhibitors (in the order of doses of each drug from the left: 0, 0.32, 1.6, 8, 40, 200, 1000 nM) in the presence of anti-CD3/CD28 activators. After 3 days, the expression of CXCR3 on (**A**) CD3, (**B**) CD4, and (**C**) CD8 T cells was determined by flow cytometry.

To identify the top candidates whose effects on GvHD will be further tested in vivo, we next screened the WU derivatives by evaluating their functional inhibitory activity on the expression of CXCR3 in primary murine T cells activated by anti-CD3/CD28 beads. CXCR3 is a chemokine receptor and surrogate marker of IFNGR-JAK1/JAK2 signaling [12,13]. Among the WU derivatives, WU4 and WU8 demonstrated significant reductions in CXCR3 expression on CD3, CD4, and CD8 T cells compared to other WU derivatives (WU1-3) in a dose-dependent manner (Figure 3), consistent with their IC_50_ (Figure 1J). The inhibitory effect of WU4 and WU8 on CXCR3 expression was comparable to other JAK inhibitors currently used in the clinic (Figure 3).

**Figure 4:**
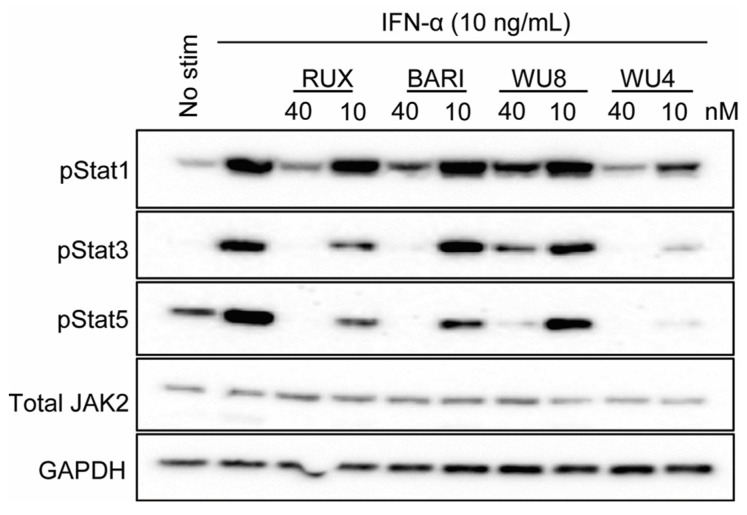
Effect of WU4 and WU8 on the inhibition of JAK signaling in primary human T cells. STAT phosphorylation was determined as the indicator of JAK signaling. Primary human T cells were obtained from the peripheral blood of healthy donors. Cells were treated with WU4, WU8, RUX, and BARI after starvation for 1 h, then stimulated with 10 ng/mL IFN-α. The phosphorylation of STATs was measured by Western blotting. Total JAK2 and GAPDH served as loading controls.

Subsequently, we examined the ability of WU4 and WU8 to inhibit JAK signaling by measuring the levels of phosphorylation of STATs, downstream transcription factors of JAK signaling. Both WU4 and WU8 inhibit the phosphorylation of STAT1, STAT3, and STAT5, with WU4 exhibiting greater potency compared to WU8 (Figure 4), suggesting that WU4 functions as an excellent pan-JAK inhibitor. This inhibitory capacity of WU4 and WU8 correlates with their IC_50_ values (Figure 1J) and the ability to reduce CXCR3 expression (Figure 3). We then decided to further investigate WU4 and WU8 as potent novel JAK inhibitors for the prevention of GvHD in our preclinical mouse model of allo-HCT.

### 2.3. The Effect of WU Derivatives on GvHD in the Preclinical Mouse Model of Allo-HCT

**Figure 5:**
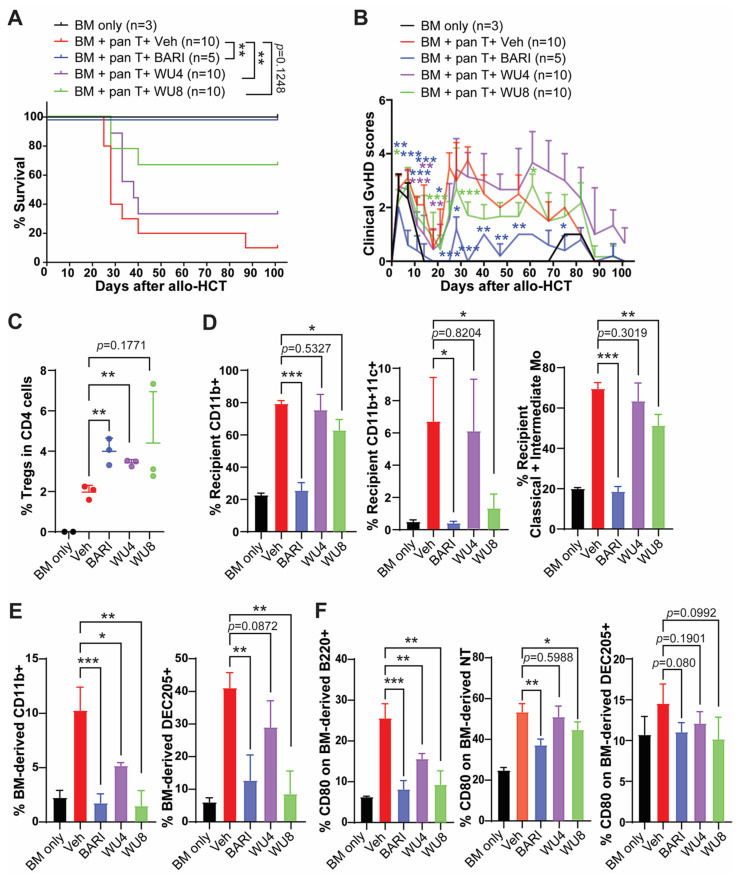
In vivo administration of WU4 and WU8 reduces GvHD by increasing % of Tregs while suppressing % of APCs and costimulatory proteins on APCs after allo-HCT. Allo-HCT was performed as follows: 5 × 10^6^ TCD-BM (CD45.1+) and 5 × 10^5^ splenic T cells (CD45.2+) obtained from B6 mice were transplanted on day 0 into lethally irradiated (900 cGy on day −1) Balb/c allogeneic recipient mice. TCD-BM-only group served as a no-GvHD control. Starting on day 3 after allo-HCT, we injected WU4, WU8, and BARI (20 mg/kg) subcutaneously once a day (5 days/week) for 3 weeks. (**A**,**B**) The mice were monitored for survival and GvHD signs. (**C**–**F**) Whole splenocytes were analyzed for % of Tregs, recipient/BM-derived APCs, and CD80-expressing APCs on day 6 after allo-HCT. LyG6^−^CD11b^+^Ly6C^high^ and LyG6^−^CD11b^+^Ly6C^middle^ served as classical and intermediate monocyte subsets. NT: neutrophils. * *p* < 0.05, ** *p* < 0.01, and *** *p* < 0.001. All error bars are represented as mean ± standard deviation.

To determine the potential of WU4 and WU8 in preventing GvHD, we performed allo-HCT in which 5 × 10^6^ T cell-depleted bone marrow (TCD-BM; CD45.1+) and 5 × 10^5^ splenic pan T cells (CD45.2+) obtained from B6 mice were transplanted into lethally irradiated (900 cGy on day −1) Balb/c allogeneic recipient mice on day 0. Mice were administered with WU4 and WU8 once daily (s.c.) at doses of 4 mg/kg or 20 mg/kg for 3 weeks, starting on day 3 after allo-HCT. Given that BARI was most effective in preventing GvHD in our previous studies, we included BARI as the reference group. Consistent with our previous reports, BARI showed a significantly improved survival rate of the recipient mice, with less clinical GvHD scores at both 4 mg/kg and 20 mg/kg after allo-HCT, throughout all experiments (Figure 5 and Figure S1A,B). We found that WU8 also significantly increased overall survival and improved clinical GvHD scores in recipients compared to the vehicle control after allo-HCT (Figure 5A,B). In addition, mice administered WU8 showed a trend towards higher frequencies of Tregs in the spleens of recipient mice on day 6 after allo-HCT compared to the vehicle control (Figure 5C). Moreover, WU8 reduced the % of both the residual recipient and donor BM-derived APCs, which can present alloantigens to donor T cells and mediate GvHD [19], compared to the control (Figure 5D,E and Figure S1C). Among monocyte subsets, we only considered classical (LyG6^−^CD11b^+^Ly6C^high^) and intermediate monocytes (LyG6^−^CD11b^+^Ly6C^middle^) as APCs, excluding non-classical monocytes (LyG6^−^CD11b^+^Ly6C^low^) due to their anti-inflammatory function, which downregulates antigen presentation during inflammation. The % of non-classical monocytes was comparable between groups (Figure S1C, right panel). The % of a co-stimulatory protein CD80-expressing donor-derived APCs (B cells, neutrophils, and dendritic cells) was also decreased in the spleens of mice administered with WU8 compared to those with the vehicle control (Figure 5F and Figure S1D). A low dose (4 mg/kg) of WU8 showed a mild trend towards improved survival and less GvHD compared to the vehicle control (Figure S1A,B), although not as much as the high dose (20 mg/kg) of the drug (Figure 5A,B). On the other hand, WU4 did not significantly improve GvHD, despite significantly increasing the % of Tregs while reducing the % of donor-derived CD11b+ APCs and CD80-expressing B cells in the spleens of mice on day 6 after allo-HCT compared to the vehicle control (Figure 5 and Figure S1A,B,D). These results indicate the significance of dual inhibition of JAK1/JAK2 while sparing JAK3 in preventing GvHD, when comparing WU4 and WU8. Thus, WU8 is a promising novel JAK inhibitor with excellent specificity for JAK1/JAK2 while sparing JAK3, representing our first-generation BARI analogue.

**Figure 6:**
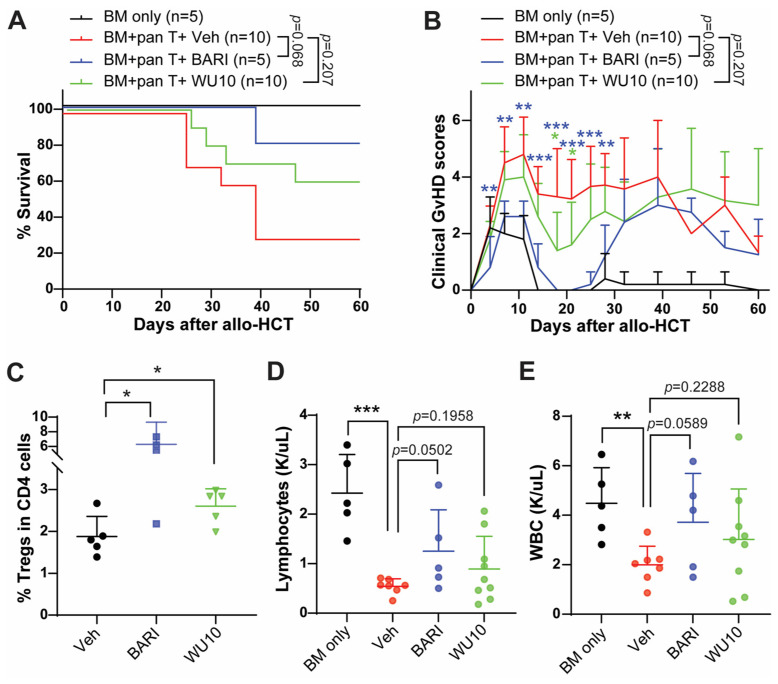
Administrating WU10 to recipient mice increased the overall survival with less GvHD by increasing Tregs and enhancing blood cell recovery. (**A**) Survival rate and (**B**) clinical GvHD scores of mice treated with WU10 after allo-HCT. (**C**) % of Tregs in the spleens of recipient mice on day 6 after allo-HCT. (**D**,**E**) Blood cell counts for lymphocytes and white blood cells using whole blood on day 27 after allo-HCT. * *p* < 0.05, ** *p* < 0.01, and *** *p* < 0.001. All error bars are represented as mean ± standard deviation.

WU10 has a nearly identical chemical structure to BARI, replacing the azetidine of BARI with a cyclobutane for WU10 (no nitrogen inside the cyclobutane) and showing similar IC_50_ to JAKs, as observed with BARI. Likewise, there was a trend towards increased overall survival with reduced GvHD in mice administrated WU10 (Figure 6A,B). Notably, WU10 significantly increased the % of Tregs in the spleens of mice on day 6 after allo-HCT compared to the vehicle control (Figure 6C). WU10 also showed a trend towards improvement in blood cell recovery (lymphocytes and white blood cells), which is negatively affected by GvHD (Figure 6D,E).

**Figure 7:**
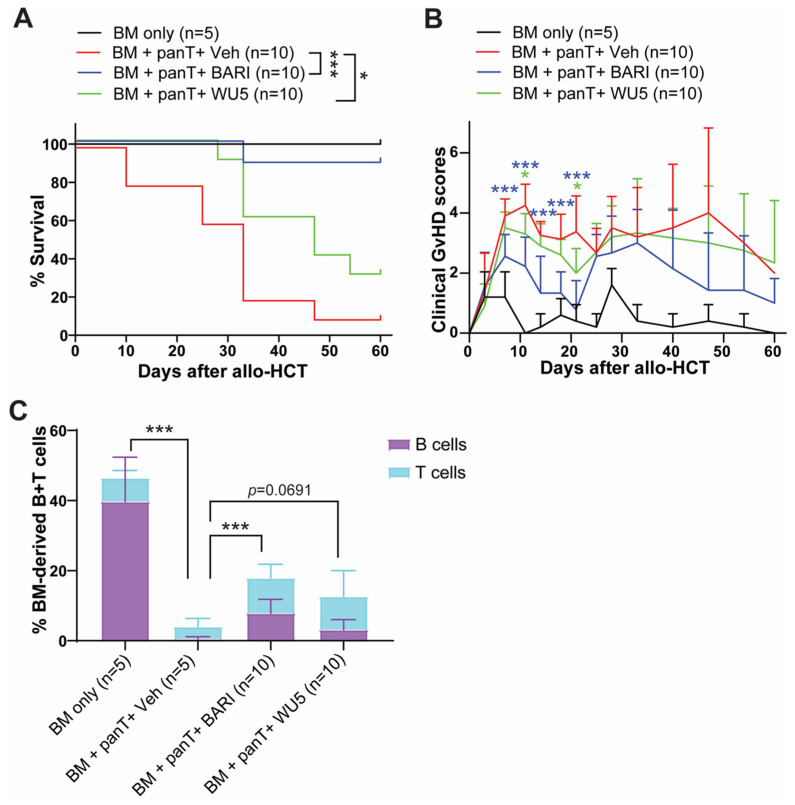
The effect of WU5 on GvHD after allo-HCT. (**A**) Survival rate and (**B**) clinical GvHD scores of mice treated with WU5 after allo-HCT. (**C**) % BM-derived B and T cells were examined in the peripheral blood on day 27 after allo-HCT. * *p* < 0.05 and *** *p* < 0.001. All error bars are represented as mean ± standard deviation.

Consistent with our previous findings, WU4 was ineffective in preventing GvHD compared to WU8 and BARI (Figure 5) due to its potent inhibitory activity against JAK3 (Figure 1J and Figure 4). Thus, we synthesized WU5 to spare JAK3 while inhibiting JAK1/JAK2, replacing the ethyl sulfonamide of WU4 with a N-t-butyl sulfamide for WU5. Sparing JAK3 in WU5 showed significantly improved overall survival with less clinical GvHD scores compared to the vehicle control (Figure 7A,B) and WU4 after allo-HCT (Figure 5A,B). In addition, WU5 increased the % of donor BM-derived B and T cells in the peripheral blood (PB) on day 27 after allo-HCT, which is consistent with an improved donor immune reconstitution and reduced GvHD compared to the vehicle control (Figure 7C). 

### 2.4. The Effect of WU6 on the Expression of CXCR3 and T-Bet in Primary Murine T Cells and GvHD in Mouse Model of Allo-HCT

**Figure 8:**
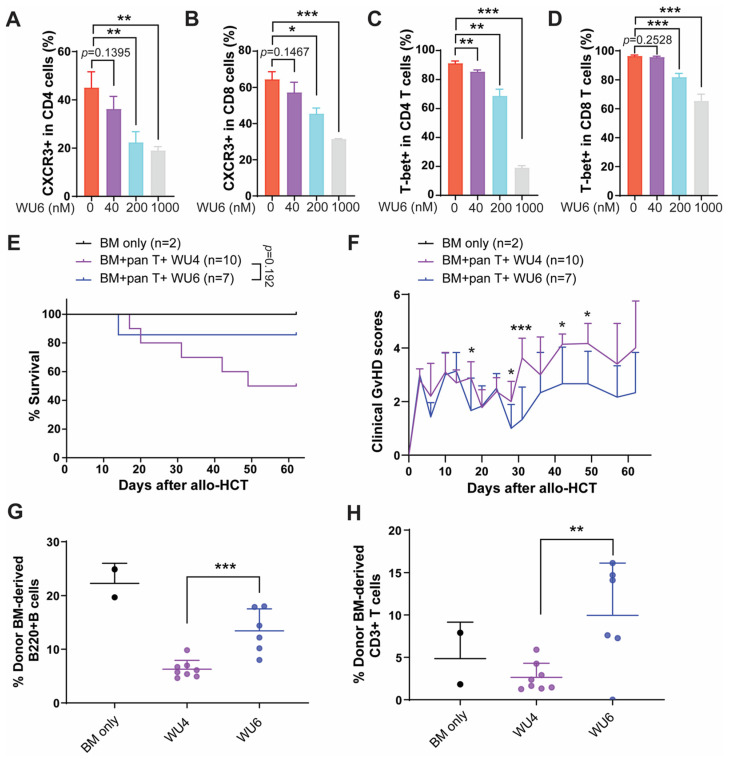
Effect of WU6 on the expression of CXCR3 and T-bet in vitro and GvHD in mouse model of allo-HCT. (**A**–**D**) Primary T cells were isolated from B6 and treated with WU6 at indicated doses for 3 days in the presence of anti-CD3/CD28 activation beads. After 3 days, the expression of CXCR3 (**A**,**B**) and T-bet (**C**,**D**) in the CD4 and CD8 was determined by flow cytometry. Survival rate and (**E**) clinical GvHD scores (**F**) of mice treated with WU6 after allo-HCT. (**G**,**H**) % BM-derived B cells (B220+) and T cells (CD3+) were examined in the peripheral blood on day 27 after allo-HCT. * *p* < 0.05, ** *p* < 0.01, and *** *p* < 0.001. All error bars are represented as mean ± standard deviation.

WU6 is a derivative of WU4, incorporating a trifluoromethane sulfonamide for WU6 instead of an ethyl sulfonamide for WU4. WU6 imparts comparable IC_50_ for both JAK1 and JAK2 (IC_50_ of JAK1 and JAK2 = 3.43 and 6.96 nM, respectively) at 1 mM of ATP compared to BARI (IC_50_ of JAK1 and JAK2 = 7.23 and 12.1 nM, respectively), effectively sparing JAK3 (IC_50_ of JAK3 = 311 nM), unlike WU4 (IC_50_ of JAK3 = 84 nM), at 1 mM of ATP. In addition, WU6 exhibits not only improved JAK3 selectivity, but also a weaker affinity to JAK3 when comparing IC_50_ values at app. Km and 1 mM of ATP compared to WU4 (Figure 1J). Subsequently, we examined the effect of WU6 on the expression of CXCR3 and T-bet (the master transcription factor of Th1 and cytotoxic T cell differentiation) in primary murine T cells. Treatment with WU6 significantly downregulated the expression of CXCR3 and T-bet in both CD4 and CD8 T cells activated by anti-CD3/CD28 activation beads in a dose-dependent manner (Figure 8 and Figure S2). In addition, sparing JAK3 in WU6 led to improved overall survival with significantly less clinical GvHD scores compared to WU4 after allo-HCT (Figure 8E,F). Moreover, WU6 significantly enhanced donor immune reconstitution, showing an increased percentage of donor-BM-derived B and T cells in the PB on day 27 after allo-HCT compared to WU4 (Figure 8G,H).

Taken together, we demonstrated that WU derivatives have the potential to improve murine GvHD by promoting Tregs and immune reconstitution while reducing both recipient- and donor-BM-derived APCs and costimulatory protein CD80 on BM-derived APCs after allo-HCT. In addition, WU derivatives efficiently decrease not only the expression of CXCR3, but also that of T-bet in both CD4 and CD8 T cells activated by anti-CD3/CD28 activation beads in vitro. The WU derivatives, especially WU8, are promising novel JAK inhibitors that could serve as alternatives to BARI or RUX.

## 3. Discussion

GvHD is a major complication after allo-HCT, significantly affecting therapeutic outcomes. The incidence of GvHD is up to 30–60%, with more than 10% of patients dying from this complication [20,21,22]. The IFNGR-JAK signaling pathway is considered a promising therapeutic target for patients with GvHD, since various JAK inhibitors have proven effective in preclinical mouse models of GvHD and clinical trials [23,24]. We were the first to report that upregulated IFNGR signaling in activated T cells contributes to GvHD development, and its genetic deletion in donor T cells significantly improves overall survival and GvHD without compromising GvL effects in vivo [12,13,25]. In addition, pharmacologic inhibition of IFNGR and IL-6R signaling using balanced JAK1/JAK2 inhibitors, such as RUX or BARI, reduces GvHD, as observed in the recipient mice transplanted with *Ifngr1*^−/−^ T cells along with anti-IL6R blocking antibody [12]. Mechanistically, blocking IFNGR/IL-6R-JAK1/JAK2 signaling alters donor T cell trafficking away from GvHD target organs and promotes preferential T cell differentiation toward Tregs and Th2 cells over Th1 cells. In addition, targeting IFNGR/IL-6R-JAK1/JAK2 signaling decreases allogeneic antigen presentation and co-stimulatory molecule expressions on APCs. Furthermore, inhibition of JAK1/JAK2 using BARI promotes gastrointestinal repair by upregulating EGFR signaling, thereby treating established GvHD after allo-HCT [15]. More recently, we demonstrated that a blockade of IFNGR signaling upregulates S100A9 expression in donor T cells, not only altering donor T cell trafficking but also modulating intestinal microbiota reconstitution after allo-HCT [25,26,27]. Surprisingly, targeting IFNGR signaling preserved or enhanced GvL effects in our preclinical mouse model of allo-HCT [12,13,27]. Since none of the strategies against GvHD have been shown to be effective in maintaining GvL effects in the clinic over the past five decades, there is an unmet medical need for novel therapeutic approaches for patients unresponsive to corticosteroids, which are the standard first-line agents for GvHD. Nonetheless, RUX is the only approved JAK inhibitor for acute and chronic GvHD for patients with steroid-resistant or refractory GvHD [28]. In addition, JAK inhibitors need to be improved in terms of safety to minimize the risk of serious side effects, such as cardiovascular conditions, blood clots, and serious infections. Therefore, developing a broad range of novel JAK inhibitors is necessary in order to minimize adverse events while still providing sufficient beneficial efficacy in preventing GvHD after allo-HCT.

**Figure 9:**
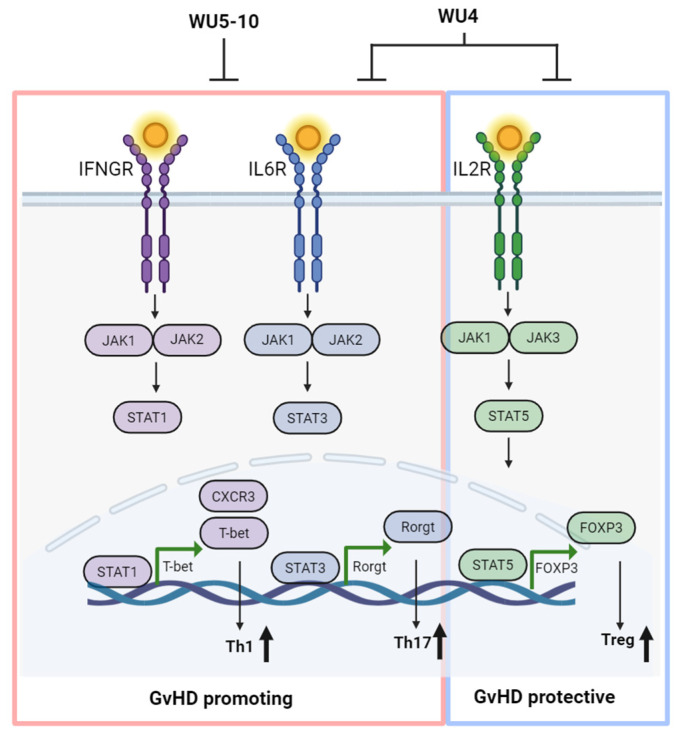
Overall schema of optimal GvHD prevention. The strategy involves inhibiting JAK1/JAK2 signaling responsible for Th1 and Th17 cells while sparing JAK3 for Tregs to achieve optimal GvHD prevention, creating WU derivatives as alternative agents for commercially available JAK inhibitors. Created with BioRender.com (accessed on 1 April 2024).

We generated novel JAK inhibitors using BARI as a scaffold structure, altering the diversity region while maintaining the core hinge binding region. The WU derivatives demonstrated significant dual inhibition against JAK1/JAK2, while some showed notable selectivity against JAK3 (Figure 9). In addition, WU derivatives demonstrated improved or comparable PK to RUX and BARI. Our results suggest potential for the WU derivatives to improve overall survival with less GvHD in our preclinical model of allo-HCT. For example, WU6 and WU8 significantly downregulate the expression of CXCR3 and/or T-bet in primary murine T cells. In addition, the administration of WU5, WU8, and WU10 shows a significant trend towards reduction in GvHD by enhancing Tregs and immune reconstitution while suppressing both recipient- and donor-BM-derived APCs, as well as costimulatory protein CD80, on the BM-derived APCs compared to the vehicle control after allo-HCT. Interestingly, WU4 significantly increased the percentage of Tregs compared to the vehicle control in our mouse model of allo-HCT, despite its powerful pan-JAK inhibitory activity. Nonetheless, WU4 failed to provide any survival benefits, suggesting that sparing JAK3 might be essential for optimal GvHD prevention regardless of the frequencies of Tregs. In addition, it is possible that WU4 can increase the frequency of Tregs independently of JAK3-STAT5 signaling. Furthermore, WU10 has a nearly identical chemical structure to BARI, replacing only the azetidine of BARI with a cyclobutane in WU10, providing a more facile and efficient synthetic method. Although WU10 shows a highly similar IC_50_ profile to BARI to a significant degree, showing potent JAK1/JAK2 inhibition while sparing JAK3, it exhibits a stronger affinity to JAK3 compared to BARI, as indicated by the ratio of IC_50_ values from ATP at app. Km to 1 mM. It is conceivable that lower binding affinity to JAK3 with the IC_50_ profile (potent JAK1/JAK2 inhibition while sparing JAK3) may affect in vivo GvHD responses, possibly contributing to BARI’s superiority over other JAK inhibitors. We also believe that the cause of the superiority of BARI to all other JAK inhibitors, including WU derivatives, is multifactorial and may arise from off-target effects. We recently found that BARI’s off target, PKN1, is inhibited by BARI, but not by RUX, in both murine and human whole-kinome analyses [29]. Moreover, plasma protein binding may also affect WU derivatives’ efficacy compared to BARI, since BARI has significantly lower plasma protein binding compared to RUX in humans [16,17]. If this is the case, the availability of WU derivatives could be improved by administering higher doses or using continuous drug-delivery techniques [30,31]. Thus, further investigation into BARI’s therapeutic benefits would involve testing dual JAK1/JAK2 inhibition while sparing JAK3, in addition to achieving improved PK and low binding affinity to JAK3 with PKN1 inhibition. In summary, although WU derivatives need to be refined in several areas to achieve complete GvHD prevention and evaluate the adverse effects in clinical trials, we demonstrate in this study that WU derivatives might be able to serve as alternative agents to commercially available JAK inhibitors.

## 4. Materials and Methods

### 4.1. JAK Inhibitor Compounds

The clinical JAK inhibitors—BARI, RUX, and additional JAK inhibitors—referenced in Figure 3 were obtained from Selleckchem and MedChemExpress. Synthetic methods for WU derivatives are as described in Supplemental Information.

### 4.2. Z’-LYTE Enzyme Activity Assay

The IC_50_ values of BARI, RUX, and WU derivatives were determined using the Z’-LYTE enzyme activity assay conducted by SelectScreen Kinase Profiling at ThermoFisher. The greater difference in IC_50_ between apparent (app.) Km and 1 mM (saturated concentration of ATP) indirectly suggests a lower binding affinity of the ATP-competitive drug to the target kinase. Further details of the assay and protocols are available online at thermofisher.com (https://www.thermofisher.com/us/en/home/industrial/pharma-biopharma/drug-discovery-development/target-and-lead-identification-and-validation/kinasebiology/kinase-activity-assays/z-lyte.html, accessed on 22 June 2018).

### 4.3. Mice

BALB/c (CD45.2+, H-2^d^), C57BL/6 (B6, CD45.1+ or CD45.2+, H-2^b^) mice were purchased from Jackson Laboratory (Bar Harbor, ME, USA). All mice were maintained in a specific pathogen-free condition facility with accessible food and water ad libitum. In the current study, we used age-matched 7-week-old male mice. Mice were cared for according to the animal welfare regulations of the Washington University School of Medicine Animal Studies Committee, and the animal study protocol was approved by the Institutional Animal Care and Use Committee (IACUC).

### 4.4. PK Studies

Mouse PK and in vitro human liver microsome (HLM) assay studies were conducted by Charles River Laboratories, Inc. (Wilmington, MA, USA). Briefly, male CD-1 mice were used in mouse PK studies. Compounds (BARI, RUX, or WU derivatives) were subcutaneously administrated at a dose of 2 mg/kg, and serial blood samples were collected at 0, 0.25, 0.5, 1, 2, 4, 6, and 24 h post-dose, with plasma compound concentrations subsequently calculated. At 24 h, no drug levels were detected in plasma for any of the compounds tested. In addition, WU derivatives were evaluated in an in vitro HLM assay to determine the intrinsic human liver microsomal clearance values of compounds in incubations with human liver microsomes.

### 4.5. Preclinical Mouse Model of Allo-HCT

Allo-HCT was performed as previously described [12,13,15,25,26,27,32]. Briefly, BALB/c mice were used as recipients and received 9 Gy total body irradiation on day −1, then were transplanted with 5 × 10^6^ T cell-depleted bone marrow (TCD-BM) cells from B6 (CD45.1+) mice along with 0.5 × 10^6^ splenic T cells from B6 (CD45.2+) mice on day 0. Mouse TCD-BM and splenic T cells were prepared using an EasySep mouse CD90.2 positive selection kit II and an EasySep mouse T cell isolation kit (STEMCELL Technologies, Cambridge, MA, USA), respectively.

### 4.6. Assessment of Clinical GvHD in Transplanted Mice

GvHD clinical scores were assessed twice per week by a scoring system incorporating five clinical criteria: weight loss (diarrhea scored 1 regardless of weight change), posture (hunching), mobility, fur texture, and skin integrity, as previously described [33]. Individual mice were scored from 0 to 2 for each criterion and were sacrificed with GvHD scores of 6 or above, following our approved animal protocol.

### 4.7. Flow Cytometry Analysis

T cells from in vitro primary murine T cell culture, fresh peripheral blood, or whole splenocytes were analyzed according to cell surface and intracellular markers. Surface markers were directly stained with flow antibodies for 20 min at 4 °C. For intracellular staining (FOXP3 and T-bet), cells were fixed and permeabilized after surface staining using appropriate buffers (One-step intracellular nuclear protein staining protocol, eBioscience, San Diego, CA, USA) and stained with flow antibodies. The antibodies used for flow cytometric analyses for mouse cells were as follows: FOXP3, T-BET, and CD183 (eBioscience, San Diego, CA, USA); CD3, H2-Kb, H2-Kd, CD4, B220, CD11b, CD11c, Ly6C, and DEC205 (BioLegend, San Diego, CA); and CD8, CD45.2, CD80, and Ly6G (BD Pharmingen, San Jose, CA, USA). All cells were run on a ZE5 Cell Analyzer (BD Biosciences, Mountain View, CA, USA) or Attune^®^ NxT (Thermofisher Scientific, Waltham, MA, USA) and analyzed with the FlowJo software, version 10.9.0 (FlowJo LLC., Ashland, OR, USA).

### 4.8. Primary Murine T Cell Culture

Primary murine T cells were isolated from B6 mice using an EasySep mouse T cell isolation kit (STEMCELL Technologies, Cambridge, MA, USA) and cultured in Xcyte media supplemented with 10U/mL IL-2 in the presence of anti-CD3/CD28 activation beads with beads/cell ratio of 1:1 (Thermofisher Scientific, Waltham, MA, USA). Cells were expanded for 3 days with/without WU derivatives, and the effect of WU derivatives on the expression of CXCR3 and T-bet was examined.

### 4.9. Statistical Analysis

The differences in the survival of the groups were determined using the log-rank test. For all other analyses, the unpaired *t*-test was used. *p*-values of less than 0.05 were considered statistically significant. All the analyses were performed using the GraphPad Prism software 10.2.2 (San Diego, CA, USA).

## Data Availability

The data presented in this study are contained within the article and are also available upon request from the corresponding author.

## Supplementary Materials

The following supporting information can be downloaded at: https://www.mdpi.com/article/10.3390/molecules29081801/s1, Figure S1: Effect of WU4 and WU8 on GvHD after allo-HCT; Figure S2: Effect of WU6 on the expression of CXCR3 and T-bet in vitro. Supplementary Information: Synthetic methods for WU derivatives.
