# Supplementary material for: Novel JAK Inhibitors to Reduce Graft-Versus-Host Disease after Allogeneic Hematopoietic Cell Transplantation in a Preclinical Mouse Model
Source: Molecules. 2024 Apr 16;29(8):1801. doi: 10.3390/molecules29081801 (PMC11052071; doi:10.3390/molecules29081801)
Supplement: Supplementary file 1 [file molecules-29-01801-s001.zip › Supplemental Information.pdf]

Supplementary Information

General methods for synthesis of WU derivatives

The starting materials were obtained from commercial sources and used without further purification after verifying their purities through LC-MS analysis. Solvents were analytical-grade and used as supplied. Non-commercially available starting materials were synthesized following the procedures in the literature, and were used after further purification and verification of their purities by ^{1}H NMR and LC-MS analysis.

Analytical HPLC analyses were performed on an Agilent 1100 system, and LC-MS analyses were conducted on Agilent 1100 Series LC/MSD (G1946C) single-quadrupole mass spectrometer system equipped with an electrospray ionization (ESI) source. Reverse-phase preparative HPLC purifications were performed either on a Biotage SP4 HPFC system or on a CombiFlash*Rf* (Teledyne Isco) system using a variable dual-wavelength UV detector on a Biotage KP-C18-HS 120 g SNAP column and on Redisep Rf Gold C18 cartridges using acetonitrile/water gradient containing 0.05% TFA. Normal-phase preparative HPLC purifications were performed either on a Biotage SP4 HPFC system or on a CombiFlash*Rf* (Teledyne Isco) system using a variable dual-wavelength UV detector with pre-packed Biotage KP-SIL SNAP cartridges, Redisep Rf silica gel (Isco) cartridges, and ethyl acetate/hexanes gradients.

All final compounds were analyzed via analytical RP-HPLC using a C18 analytical column with a diode array detector, and peaks were monitored at 210, 254, and 280 nM for their purity. ^{1}H and ^{19}F NMR spectra were recorded in deuterated solvents (DMSO-d_6, CD_3OD, and $CDCl_3$) on a

Varian 400 MHz NMR spectrometer equipped with a Broad Band NMR probe and an autosampler. The signal of the deuterated solvent was used as an internal reference. The chemical shifts are expressed in ppm (δ), and coupling constants (J) are reported in hertz (Hz). The following abbreviations are used to express the multiplicities: s = singlet; d = doublet; t = triplet, q = quartet; dd = doublet of doublets; m = multiplet; and br = broad. Reactions were performed under an atmosphere of dry nitrogen unless otherwise stated.

Synthetic methods for WU derivatives

1. **WU4,** N-(6-(4-(7H-pyrrolo[2,3-*d*]pyrimidin-4-yl)-1*H*-pyrazol-1-yl)-6-(cyanomethyl) spiro[3.3]heptan-2-yl) ethanesulfonamide.

Synthetic Scheme for the Synthesis of WU4:

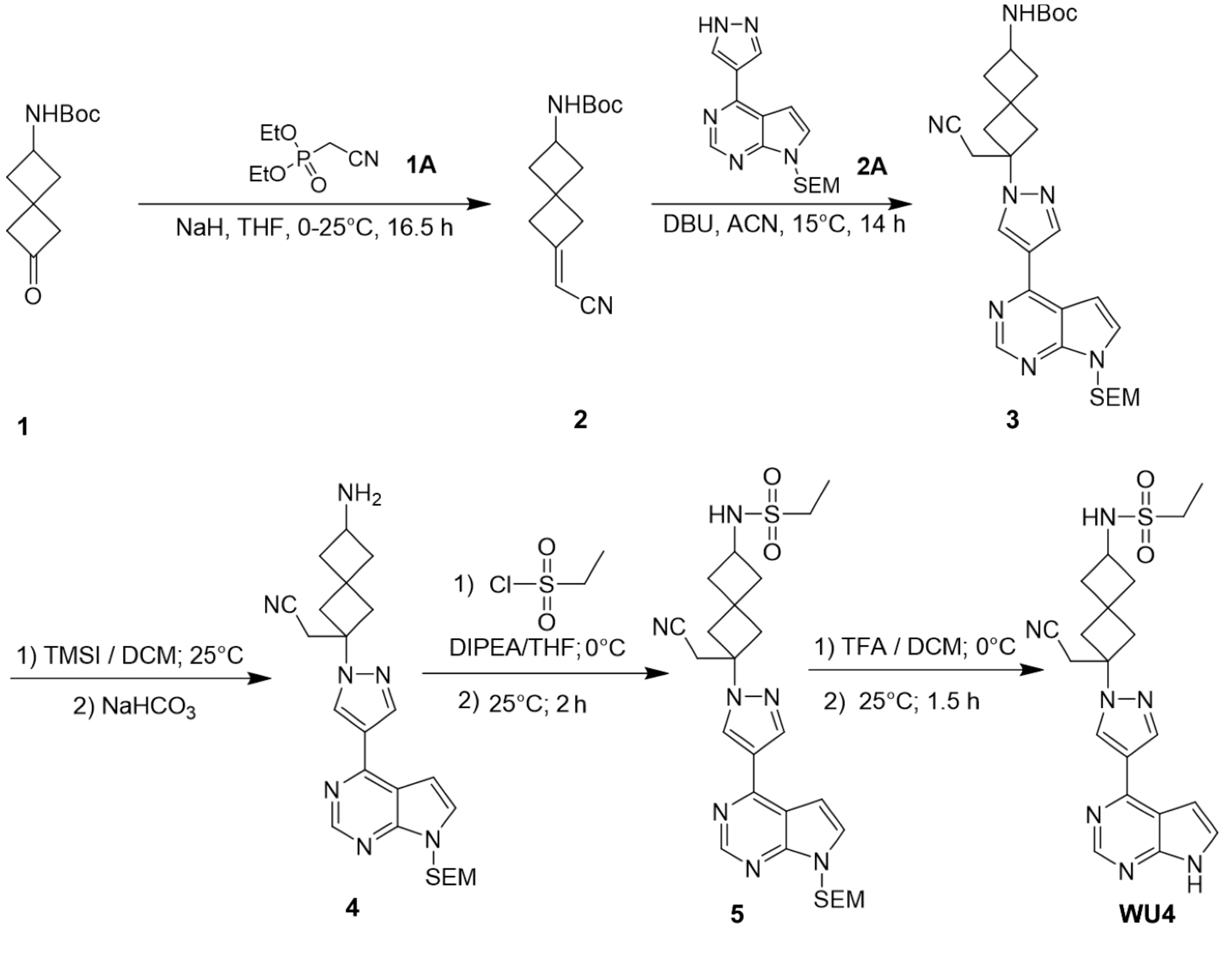

Step 1. Preparation of *tert*-butyl (6-(cyanomethylene)spiro[3.3]heptan-2-yl)carbamate.

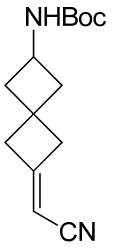

2

To a solution of NaH (2.13 g, 53.2 mmol, 60% purity, 1.20 *eq*) in THF (100 mL), diethyl (cyanomethyl) phosphonate **(1A)** (9.04 g, 51.0 mmol, 8.22 mL, 1.15 *eq*) was added at 0°C under an N_2 atmosphere. Then, the mixture was stirred at 25°C for 45 min. Then, 2-(Boc-amino)-6-oxospiro[3.3]heptane **(1)** Supplier: Combi-Blocks, cat. # SS-1671 (10.0 g, 44.3 mmol, 1.00 *eq*), in THF (100 mL) was added drop-wise. The mixture was stirred at 25°C for 16 h. The mixture was poured into water (100 mL) and then extracted with ethyl acetate (100 mL x 3). The combined organic phase was washed with brine (100 mL) and the combined organic layers were dried over Na_2SO_4, concentrated under vacuum. The crude product was triturated with (Petroleum ether/Ethyl acetate = 50/1) at 25°C for 15 min. The mixture was filtered, and the filter cake was concentrated under vacuum to obtain *tert*-butyl (6-(cyanomethylene)spiro[3.3]heptan-2-yl)carbamate **(2)** (9.10 g, 36.6 mmol, 82.5% yield) as a yellow solid. ^{1}H NMR (400 MHz, $CDCl_3$): δ 5.13 - 5.17 (m, 1H), 4.66 (s, 1H), 4.04 - 4.06 (m, 1H), 2.82 - 3.02 (m, 4H), 2.46 - 2.49 (m, 2H), 1.96 - 1.99 (m, 2H), 1.44 (s, 9H).

Step 2. Preparation of *tert*-butyl (6-(cyanomethyl)-6-(4-(7-((2-

(trimethylsilyl)ethoxy)methyl)-7*H*-pyrrolo[2,3-*d*]pyrimidin-4-yl)-1*H*-pyrazol-1-yl)spiro[3.3]heptan-2-yl)carbamate.

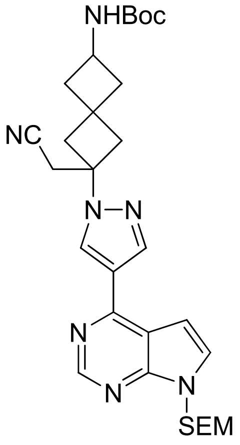

3

To a solution of tert-butyl (6-(cyanomethylene)spiro[3.3]heptan-2-yl)carbamate **(2)** (8.00 g, 32.2 mmol, 1.00 *eq*) and 4-(1*H*-pyrazol-4-yl)-7-((2-(trimethylsilyl)ethoxy)methyl)-7*H*-pyrrolo[2,3-*d*]pyrimidine **(2A) (**Supplier: Sigma Aldrich, cat. # ADE001297) (8.30 g, 26.4 mmol, 0.82 *eq*) in MeCN (90 mL), DBU (4.90 g, 32.2 mmol, 4.86 mL, 1.00 *eq*) was added. The mixture was stirred at 25°C for 16 h. The mixture was poured into water (100 mL) and extracted with ethyl acetate (100 mL x 3). The combined organic phase was washed with brine (100 mL), dried over Na_2SO_4, filtered, and concentrated. The crude product was triturated with (Petroleum ether/Ethyl acetate = 30/1) at 25°C for 15 min. The mixture was filtered, and the filter cake was concentrated under vacuum to obtain *tert*-butyl (6-(cyanomethyl)-6-(4-(7-((2-(trimethylsilyl)ethoxy)methyl)-7*H*-pyrrolo[2,3-d]pyrimidin-4-yl)-1*H*-pyrazol-1-yl)spiro[3.3]heptan-2-yl)carbamate **(3)** (14.0 g, 23.9 mmol, 74.2% yield, 96.2% purity) as a white solid. ^{1}H NMR (400 MHz, $CDCl_3$): δ 8.84 (s, 1H), 8.36 (s, 1H), 8.29 (s, 1H), 7.39 - 7.40 (d, *J* = 3.6 Hz, 1H), 6.79 - 6.80 (d, *J* = 3.6 Hz, 1H), 4.75 (s, 2H),

4.13 (s, 1H), 3.53 - 3.57 (m, 2H), 2.86 - 3.06 (m, 2H), 2.86 - 2.95 (m, 2H), 2.69 - 2.73 (m, 1H), 2.58 - 2.64 (m, 2H), 2.38 - 2.44 (m, 1H), 2.07 - 2.12 (m, 1H), 1.70 - 2.01 (m, 1H), 1.43 (s, 9H), 0.91 - 0.95 (m, 2H), -0.053 (s, 9H).

Step 3. Preparation of 2-(6-amino-2-(4-(7-((2-(trimethylsilyl)ethoxy)methyl)-7*H*-pyrrolo[2,3-*d*] pyrimidin-4-yl)-1*H*-pyrazol-1-yl)spiro[3.3]heptan-2-yl)acetonitrile.

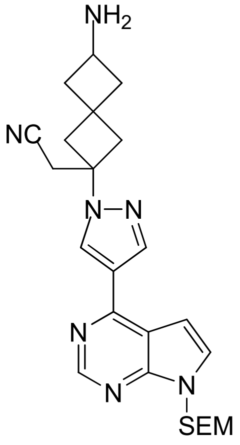

4

To a solution of *tert*-butyl (6-(cyanomethyl)-6-(4-(7-((2-(trimethylsilyl)ethoxy)methyl)-7*H*-pyrrolo[2,3-*d*]pyrimidin-4-yl)-1*H*-pyrazol-1-yl)spiro[3.3]heptan-2-yl)carbamate **(3)** (2.50 g, 4.43 mmol, 1.00 *eq*) in DCM (30 mL), iodotrimethylsilane (1.77 g, 8.87 mmol, 1.21 mL, 2.00 *eq*) was added drop-wise at 25°C. Then, the mixture was stirred at 25°C for 2 h. The mixture was adjusted with saturated aqueous $NaHCO_3$ until pH = 8 and extracted with DCM (100 mL x 3). The combined organic phase was washed with brine (100 mL) and the combined organic layers were dried over Na_2SO_4 and concentrated under vacuum

to yield 2-(6-amino-2-(4-(7-((2-(trimethylsilyl)ethoxy)methyl)-7*H*-pyrrolo[2,3-*d*]pyrimidin-4-yl)-1*H*-pyrazol-1-yl)spiro[3.3]heptan-2-yl) acetonitrile **(4)** (2.00 g, 4.27 mmol, 96.2% yield, 98.9% purity) as a yellow oil, which was used for the next step directly. ^{1}H NMR (400 MHz, $CDCl_3$): δ 8.84 (s, 1H), 8.36 (s, 1H), 8.29 (s, 1H), 7.39 - 7.41 (d, J = 3.6 Hz, 1H), 6.79 - 6.81 (d, J = 3.6 Hz, 1H), 5.68 (s, 2H), 3.53 - 3.57 (m, 2H), 3.43 - 3.47 (m, 1H), 3.07 (s, 2H), 2.84 - 2.91 (m, 2H), 2.66 - 2.70 (m, 1H), 2.56 - 2.61 (m, 2H), 2.35 - 2.37 (m, 1H), 1.91 - 1.96 (m, 1H), 1.80 - 1.83 (m, 1H), 0.91 - 0.95 (m, 2H), -0.053 (s, 9H).

Step 4. Preparation of *N*-(6-(cyanomethyl)-6-(4-(7-((2-(trimethylsilyl)ethoxy)methyl)-7*H*-pyrrolo [2,3-*d*]pyrimidin-4-yl)-1*H*-pyrazol-1-yl)spiro[3.3]heptan-2-yl)ethanesulfonamide.

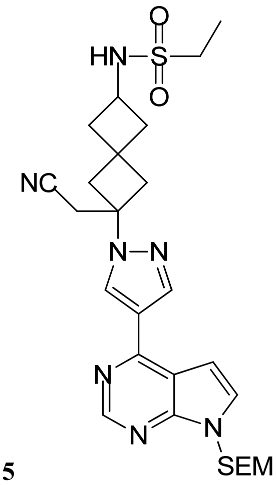

5

To a solution of 2-(6-amino-2-(4-(7-((2-(trimethylsilyl)ethoxy)methyl)-7*H*-pyrrolo[2,3-*d*]pyrimidin-4-yl)-1*H*-pyrazol-1-yl)spiro[3.3]heptan-2-yl)acetonitrile **(4)** (1.00 g, 2.16

mmol, 1.00 eq) and DIPEA (557 mg, 4.31 mmol, 751 uL, 2.00 eq) in THF (10 mL), ethanesulfonyl chloride (277 mg, 2.16 mmol, 204 uL, 1.00 eq) was added at 0°C; then, the mixture was stirred at 25°C for 2 h. The mixture was poured into water (50 mL) and extracted with ethyl acetate (50 mL x 3). The combined organic phase was washed with brine (50 mL) and the combined organic layers were dried over Na_2SO_4, concentrated under vacuum. The crude *N*-(6-(cyanomethyl)-6-(4-(7-((2-(trimethylsilyl)ethoxy)methyl)-7*H*-pyrrolo [2,3-*d*]pyrimidin-4-yl)-1*H*-pyrazol-1-yl)spiro[3.3]heptan-2-yl)ethanesulfonamide **(5)** (1 g, crude) was used for the next step directly.

Step 5. Preparation of *N*-(6-(4-(7*H*-pyrrolo[2,3-*d*]pyrimidin-4-yl)-1*H*-pyrazol-1-yl)-6-(cyanomethyl) spiro[3.3]heptan-2-yl)ethanesulfonamide (WU4).

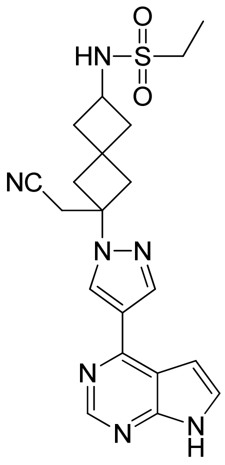

WU4

To a solution of *N*-(6-(cyanomethyl)-6-(4-(7-((2-(trimethylsilyl)ethoxy)methyl)-7*H*-pyrrolo[2,3-*d*]pyrimidin-4-yl)-1*H*-pyrazol-1-yl)spiro[3.3]heptan-2-yl)ethanesulfonamide **(5)** (400 mg, 899 umol, 1.00 eq) in DCM (10 mL), TFA (3.85 g, 33.7 mmol, 2.50 mL, 37.5 eq) was added drop-wise at 0°C. The mixture was stirred at 25°C for 1.5 h. The mixture was concentrated in vacuum, the residue was dissolved in methanol (10 mL), and NH_3H_2O (3 mL) was added to the mixture; then, the mixture was stirred at 25°C for 3 h. The mixture was added to H_2O (10 mL) and extracted with dichloromethane (10 mL x 3). The combined organic phase was washed with brine (50 mL), dried over Na_2SO_4, filtered, and concentrated. The mixture was purified using prep-HPLC (column: Waters Xbridge 150x25 5u;mobile phase: [water (0.05% ammonium hydroxide v/v)-ACN]; B%: 15%-45%,10 min) to yield *N*-(6-(4-(7*H*-pyrrolo[2,3-*d*]pyrimidin-4-yl)-1*H*-pyrazol-1-yl)-6-(cyanomethyl) spiro[3.3]heptan-2-yl)ethanesulfonamide **(WU4)** (209 mg, 490 umol, 54.5% yield, 99.8% purity), which was obtained as an off-white solid. LC-MS analysis of the solid confirmed the product mass: m/z 426.2 (M+H)+: Calculated for $C_{20}H_{23}N_7O_2S$ = 425.16 ^1H NMR (400 MHz, $CDCl_3$): δ 9.58 - 9.60 (m, 1H), 8.82 (d, *J* = 5.2 Hz, 1H), 8.38 (s, 1H), 8.31 (s, 1H), 7.38 - 3.39 (m, 1H), 6.79 - 6.80 (m, 1H), 4.62 - 4.65 (m, 1H), 3.87 - 3.97 (m, 1H), 3.01 - 3.05 (m, 2H), 2.95 - 2.99 (m, 4H), 2.72 - 2.75 (m, 3H), 2.40 - 2.52 (m, 1H), 2.11 - 2.20 (m, 1H), 2.08 - 2.09 (m, 1H), 1.35 (t, *J* = 7.2 Hz, 3H).

The additional compounds were synthesized via the same procedure as WU4, merely substituting the appropriate ketone, as described for each below, or the appropriate sulfonyl

chloride, depending upon the analogue described below.

2. WU1, 2-(6-(4-(7H-pyrrolo[2,3-d]pyrimidin-4-yl)-1H-pyrazol-1-yl)-2-(ethylsulfonyl)-2-azaspiro[3.3]heptan-6-yl)acetonitrile.

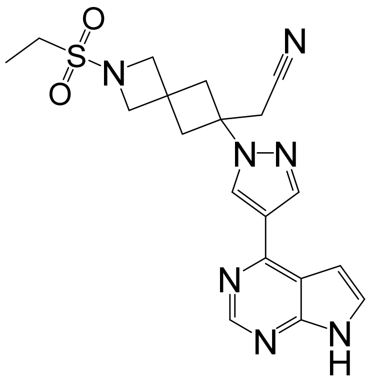

WU1 was synthesized as described above for the synthesis of WU4, but substituting *tert*-Butyl 6-oxo-2-azaspiro[3.3]heptane-2-carboxylate (Supplier: Combi-Blocks cat. # SS-1643) for 2-(Boc-amino)-6-oxospiro[3.3]heptane (1) in Step 1. The crude product was purified via reverse-phase preparative HPLC to afford WU1 as a colorless solid, with a 90% yield. LC-MS analysis of the solid confirmed the product mass: m/z 412 $[M+H]^+$; m/z 434 $[M+Na]^+$. It was also calculated for $C_{19}H_{21}N_7O_2S$: 411.48 ^{1}H NMR (400 MHz, CD_3OD): δ 8.89 (s, 1H), 8.86 (s, 1H), 8.49 (s, 1H), 7.82 (d, J = 3.50 Hz, 1H), 7.29 (d, J = 3.50 Hz, 1H), 4.09 (s, 1H), 3.91 (s, 1H), 3.27 (s, 2H), 3.15 (d, J = 14.00 Hz, 2H), 3.02 (q, J = 7.40 Hz, 2H), 2.89 (brd, J = 14.00 Hz, 2H), 1.35 (dd, J = 6.62, 3.50 Hz, 3H), 1.28 (t, J = 7.40 Hz, 3H).

3. **WU2,** 2-(6-4-(7H-pyrrolo[2,3-d]pyrimidin-4-yl)-1H-pyrazol-1-yl)-1-(ethylsulfonyl)-1-azaspiro[3.3]heptan-6-yl)acetonitrile.

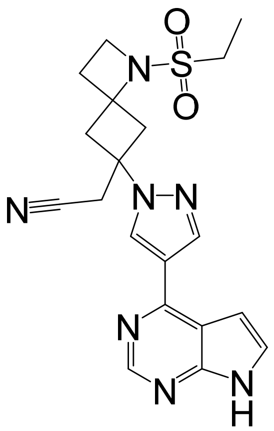

WU2 was synthesized as described above for the synthesis of WU4, but substituting *tert*-butyl 6-oxo-1-azaspiro[3.3]heptane-1-carboxylate (Supplier: 1Click Chemistry cat. # 2C24063) for 2-(Boc-amino)-6-oxospiro[3.3]heptane (1) in Step 1. The crude product was purified via reverse-phase preparative HPLC to afford WU2 as a colorless solid, with a 47% yield. LC-MS analysis of the solid confirmed the product mass: m/z 412.0 $(M+H)^+$; m/z 434.0 $(M+Na)^+$. It was also calculated for $C_{19}H_{21}N_7O_2S$ = 411.48 ^1H NMR (400 MHz, CD_3OD): δ 8.91 (s, 1H), 8.88 (s, 1H), 8.50 (s, 1H), 7.84 (s, 1H), 7.37 (s, 1H), 3.68-3.84 (m, 2H), 3.44-3.62 (m, 2H), 2.97 (q, J = 7.66 Hz, 2H), 2.90-2.93 (m, 2H), 2.66 (s, 2H), 2.43 (t, J = 7.01 HZ, 2H), 1.38 (t, J =7.01 Hz, 2H), 1.24 (t, J = 7.40 Hz, 3H).

4. WU3, 2-(5-(4-(7H-pyrrolo[2,3-d]pyrimidin-4-yl)-1H-pyrazol-1-yl)-2-(ethylsulfonyl)-2-azaspiro[3.3]heptan-5-yl)acetonitrile.

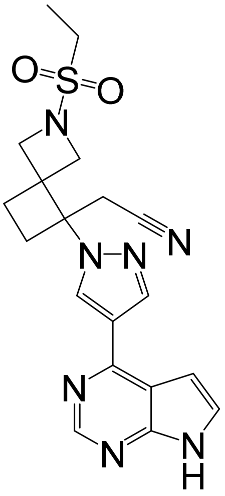

WU3 was synthesized as described above for the synthesis of WU4, but substituting *tert*-butyl 5-oxo-2-azaspiro[3.3]heptane-2-carboxylate (Supplier: 1Click Chemistry cat. # 4C12038) for 2-(Boc-amino)-6-oxospiro[3.3]heptane (1) in Step 1. The crude product was purified by reverse-phase preparative HPLC to afford WU3 as a cream solid with a 40.3% yield. LC-MS analysis of the solid confirmed the product mass: m/z 412 $[M+H]^+$; m/z 434 $[M+Na]^+$. It was also calculated for $C_{19}H_{21}N_7O_2S$: 411.48 ^1H NMR (400 MHz, CD_3OD): δ 8.86 (s, 1H), 8.85 (s, 1H), 8.44 (s, 1H), 7.82 (d, J = 3.89 Hz, 1H), 7.26 (d, J = 3.89 Hz, 1H), 4.09-4.32 (m, 2H), 3.11 (q, J = 7.40 Hz, 2H), 2.66-2.99 (m, 2H), 2.05-2.42 (m, 4H), 1.23-1.48 (m, 6H).

5. WU5, N-(6-(4-(7H-pyrrolo[2,3-d]pyrimidin-4-yl)-1H-pyrazol-1-yl)-6-(cyanomethyl)spiro[3.3]heptan-2-yl)-tert-butylsulfamoylamide.

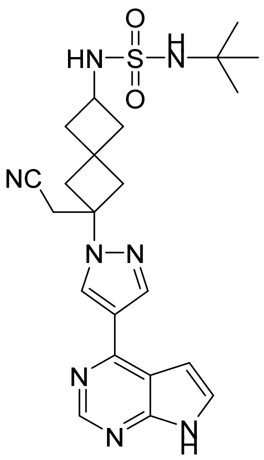

WU5 was synthesized as described above for the synthesis of WU4, but substituting *tert*-butylsulfamoyl chloride (Supplier: 1Click Chemistry cat. #1C18375) for ethanesulfonyl chloride in Step 4. The crude product was purified using pre-HPLC (column: Xtimate C18 10 u 250 mm*80 mm; mobile phase: [water (0.05% ammonium hydroxide v/v)-ACN]; B%: 26%-46%, 10 min) to yield WU5 as a white solid, with a 7.38% yield. LC-MS analysis of the solid confirmed the product mass: m/z 469.4 $(M+H)^+$. It was also calculated for $C_{22}H_{28}N_8O_2S$ = 468.2 ^{1}H NMR: (400 MHz DMSO-d_6): δ 8.70 (d, J = 10.8 Hz, 2H), 8.39 (s, 1H), 7.59 (d, J = 3.2 Hz, 1H), 7.05 (d, J = 3.6 Hz, 1H), 6.48 (s, 1H), 3.60 (s, 1H), 2.91 (d, J = 12.8 Hz, 3H), 2.60 (s, 3H), 2.45 - 2.46 (m, 1H), 2.22 - 2.24 (m, 1H), 2.10 - 2.15 (m, 1H), 2.00 - 2.05 (m, 1H), 1.20 (s, 9H).

6. **WU6,** N-(6-(4-(7H-pyrrolo[2,3-d]pyrimidin-4-yl)-1H-pyrazol-1-yl)-6-(cyanomethyl)spiro [3.3]heptan-2-yl)-1,1,1-trifluoromethanesulfonamide.

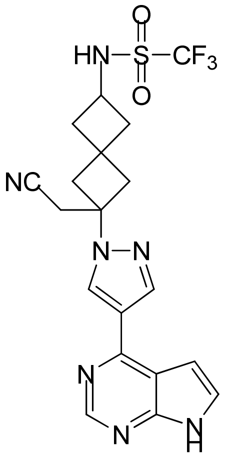

WU6 was synthesized as described above for the synthesis of WU4, but substituting trifluoromethanesulfonyl chloride (Supplier: Sigma Aldrich cat. # 164798) for ethane sulfonyl chloride in Step 4, and was purified by pre-HPLC (column: Phenomenex Gemini 150*25 mm*10 um;mobile phase: [water (10 mM NH_4HCO_3)-ACN]; B%: 18%-48%,10 min) to yield WU6 as an off-white solid, with a 31.3% yield. LC-MS analysis of the solid confirmed the product mass: m/z 466.3 $(M+H)^+$. It was also calculated for $C_{19}H_{18}F_3N_7O_2S$ = 465.12 ^1H NMR (400 MHz, DMSO-d_6): δ 12.10 (d, J = 0.73 Hz, 1H), 9.72 (br s, 1H), 8.70 (s, 1H), 8.68 (s, 1H), 8.38 (s, 1H), 7.59 - 7.60 (m, 1H), 7.05 (dd, J = 3.55,1.17 Hz, 1H), 3.87 (t, J = 8.07 Hz, 1H), 2.86 - 2.97 (m, 2H), 2.66 - 2.67 (m, 2H), 2.52 (br s, 4H), 2.16 - 2.23 (m, 1H), 2.06 - 2.13 (m, 1H).

7. **WU7,** 2-(9-(4-(7H-pyrrolo[2,3-d]pyrimidin-4-yl)-1H-pyrazol-1-yl)-3-(ethylsulfonyl)-3-azaspiro[5.5]undecan-9-yl)acetonitrile.

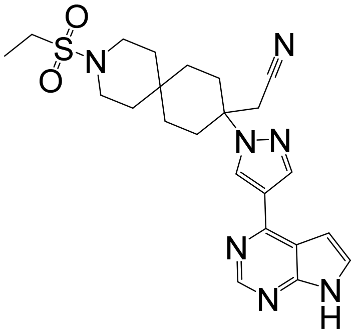

WU7 was synthesized as described above for the synthesis of WU4, but substituting *tert*-Butyl 9-oxo-3-azaspiro[5.5]undecane-3-carboxylate (Supplier: Combi-Blocks cat. # QA-9832) for 2-(Boc-amino)-6-oxospiro[3.3]heptane (1) in Step 1. The crude product was purified by reverse-phase preparative HPLC to afford WU7 as a white solid, with a 70% yield. LC-MS analysis of the solid confirmed the product mass: m/z 468 $[M+H]^+$; m/z 490 $[M+Na]^+$. It was also calculated for $C_{23}H_{29}N_7O_2S$: 467.59 ^1H NMR (400 MHz, CD_3OD): δ 8.96 (s, 1H), 8.91 (s, 1H), 8.56 (s, 1H), 7.87 (d, J = 3.89 Hz, 1H), 7.35 (d, J = 3.89 Hz, 1H), 3.29-3.34 (m, 2H), 3.21-3.27 (m, 2H), 3.15 (s, 2H), 3.03 (q, J = 7.40 Hz, 2H), 2.58-2.69 (m, 2H), 2.11-2.23 (m, 2H), 1.66-1.77 (m, 4H), 1.34-1.50 (m, 4H), 1.31 (t, J = 7.40 H, 3H).

8. WU8, 2-(4-(4-(7H-pyrrolo[2,3-d]pyrimidin-4-yl)-1H-pyrazol-1-yl)-1,1-dioxidotetrahydro-2H-thiopyran-4-yl)acetonitrile.

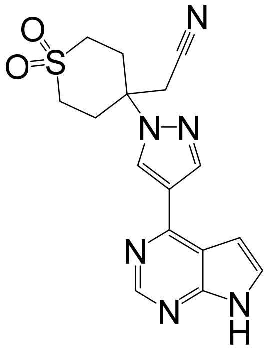

WU8 was synthesized as described above for the synthesis of WU4, but substituting 1,1-Dioxo-tetrahydro-thiopyran-4-one (Supplier: Combi-Blocks cat. # QA-0853)

for 2-(Boc-amino)-6-oxospiro[3.3]heptane **(1)** in Step 1. The crude product was purified via reverse-phase preparative HPLC (column: Biotage KP-C18 HS 120 g column; mobile phase: [water/ACN]; 10%-70%) to yield WU8 as a colorless crystalline solid, with an 80% yield. LC-MS analysis of the solid confirmed the product mass: m/z 357.0 $(M+H)^+$; m/z 379 $(M+Na)^+$. It was also calculated for $C_{16}H_{16}N_6O_2S$ = 356.40 ^{1}H NMR (400 MHz, DMSO-d_6): δ 12.63 (brs,1H, NH), 9.01 (s, 1H), 8.86 (s, 1H), 8.57 (s, 1H), 7.79 (s, 1H), 7.26 (s, 1H), 3.42 (s, 2H), 3.31 (d, J = 13.62 Hz, 2H), 3.12 (d, J = 14.40 Hz), 3.04 (t, J = 12.26 Hz, 2H), 2.59 (t, J = 12.26 Hz, 2H).

9. WU9, 2-(3-(4-(7H-pyrrolo[2,3-d]pyrimidin-4-yl)-1H-pyrazol-1-yl)-1,1-dioxidothietan-3-yl)acetonitrile.

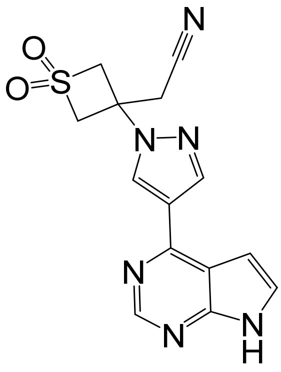

WU9 was synthesized as described above for the synthesis of WU4, but substituting Thietan-3-One 1,1-Dioxide (Supplier: AstaTech cat. # D75198) for 2-(Boc-amino)-6-oxospiro[3.3]heptane (1) in Step 1. The crude product was purified via reverse-phase preparative HPLC to afford WU9 as a white solid, with a 93.6% yield. LC-MS analysis of the solid confirmed the product mass: m/z 329.0 $(M+H)^+$; m/z 351 $(M+Na)^+$. It was also calculated for $C_{14}H_{12}N_6O_2S$ = 328.35 ^1H NMR (400 MHz, CD_3OD): δ 9.18 (s, 1H), 8.94 (s, 1H), 8.58 (s, 1H), 7.90 (d, J = 3.89 Hz, 1H), 7.37 (d, J = 3.89 Hz, 1H), 5.10-5.25 (m, 2H), 4.85-4.90 (m, 2H), 3.71 (s, 2H), 2.66 (s, 1H).

10. WU10, 2-(1-(4-(7H-pyrrolo[2,3-d]pyrimidin-4-yl)-1H-pyrazol-1-yl)-3-(methylsulfonyl) cyclobutyl)acetonitrile.

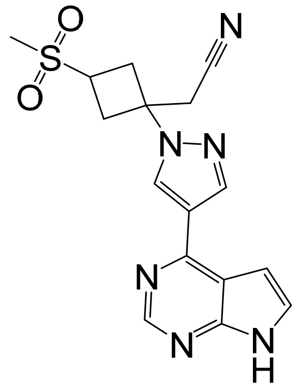

WU10 was synthesized as described above for the synthesis of WU4, but substituting 3-methanesulfonylcyclobutan-1-one (Supplier: 1Click Chemistry cat. # 4C32576) for 2-(Boc-amino)-6-oxospiro[3.3]heptane (1) in Step 1. The crude product was purified by reverse-phase preparative HPLC to afford WU10 as a white solid, with a 37% yield. LC-MS analysis of the solid showed the desired product's mass: m/z 357 $[M+H]^+$ and m/z 379 $[M+Na]^+$. It was also calculated for $C_{16}H_{16}N_6O_2S$: 356.40 ^{1}H NMR (400 MHz, CD_3OD): δ 8.97 (s, 1H), 8.87 (s, 1H), 8.50 (s, 1H), 7.82 (d, J = 3.50 Hz, 1H), 7.30 (d, J = 3.50 Hz, 1H), 4.09-4.28 (m, 2H), 3.52 (s, 2H), 3.22-3.29 (m, 2H), 2.99-3.06 (m, 2H), 2.97 (s, 3H).
