# Supplementary material for: Novel JAK Inhibitors to Reduce Graft-Versus-Host Disease after Allogeneic Hematopoietic Cell Transplantation in a Preclinical Mouse Model
Source: Molecules. 2024 Apr 16;29(8):1801. doi: 10.3390/molecules29081801 (PMC11052071; doi:10.3390/molecules29081801)
Supplement: Supplementary file 1 [file molecules-29-01801-s001.zip › Supplementary Figures.pdf]

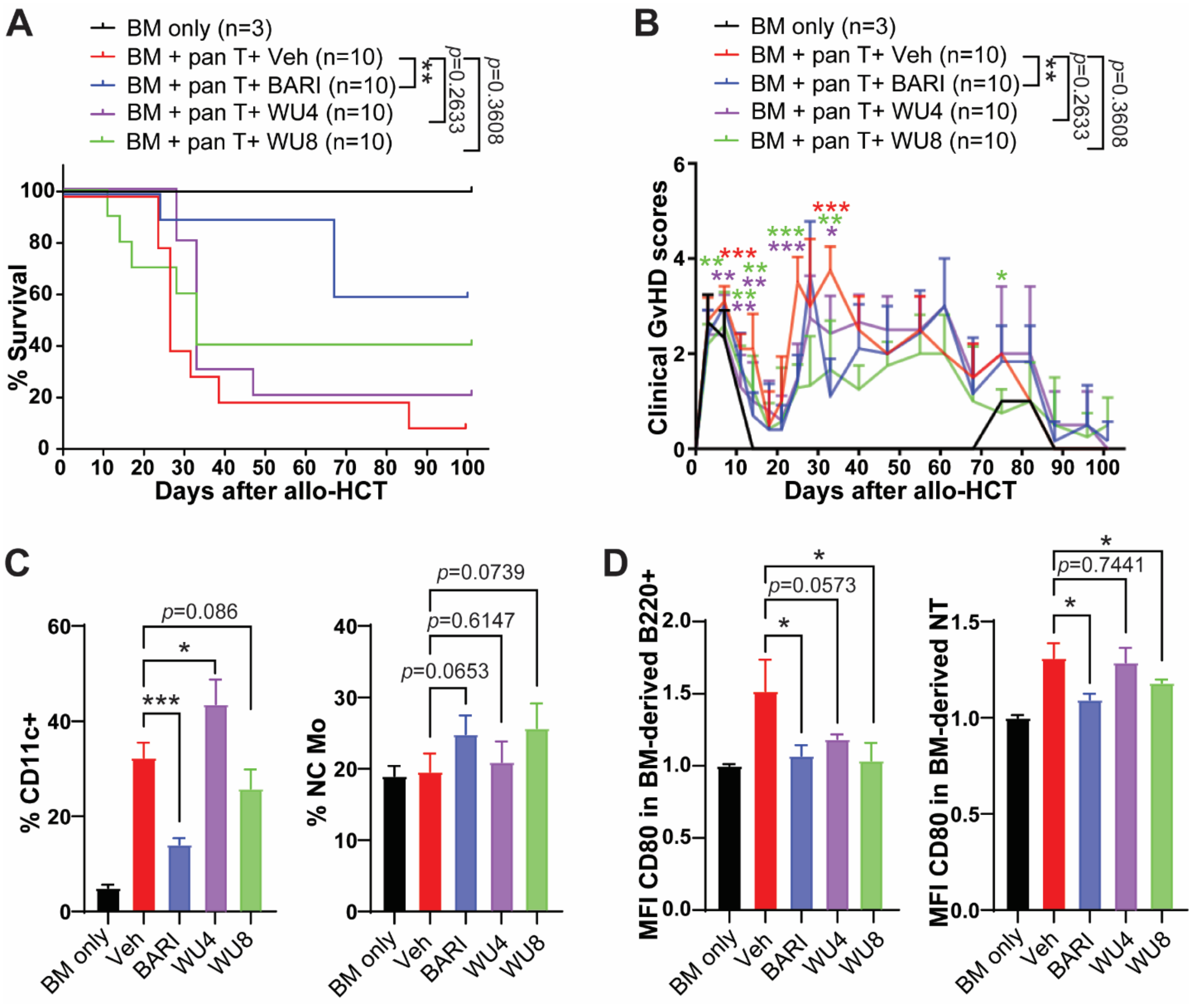

Supplementary Figure S1. Effect of WU4 and WU8 on GvHD after allo-HCT. Allo-HCT was performed as follows; 5×10^{6} TCD-BM (CD45.1+) and 5×10^{5} splenic T cells (CD45.2+) obtained from B6 mice were transplanted on day 0 into lethally irradiated (900 cGy on day -1) Balb/c allogeneic recipient mice. TCD-BM only group serves as no GvHD control. (A-B) Starting on day 3 after allo-HCT, we injected WU4, WU8, and BARI (4 mg/kg) subcutaneously once a day (5 days/week) for 3 weeks. The mice were monitored for survival and GvHD signs. (C) Whole splenocytes were analyzed for % of the recipient's APCs (CD11c+ and non-classical monocytes [NC Mo]) and (D) CD80-expressing BM-derived B cells (B220+) and neutrophils (NT) on day 6

after allo-HCT. LyG6-CD11b+Ly6C^{high} and LyG6-CD11b+Ly6C^{middle} serve as classical and intermediate monocyte subsets. *p<.05, ** p<.01, and *** p<.001. All error bars are represented as mean±standard deviation.

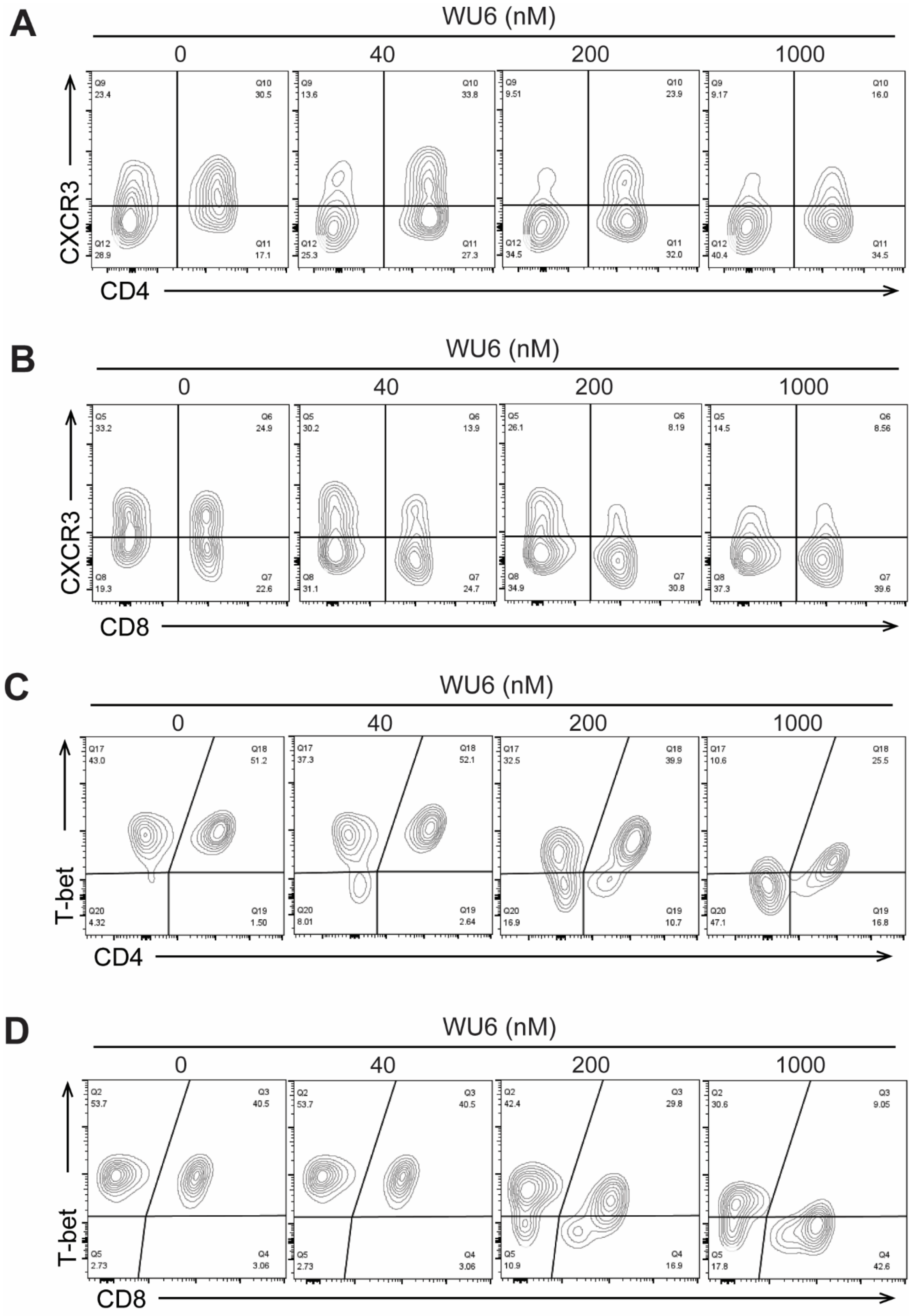

A
WU6 (nM)
0
40
200
1000
Q9 23.4
Q10 30.5
Q12 28.9
Q11 17.1
Q9 13.6
Q10 33.8
Q12 25.3
Q11 27.3
Q9 9.51
Q10 23.9
Q12 34.5
Q11 32.0
Q9 9.17
Q10 16.0
Q12 40.4
Q11 34.5
CXCR3
CD4
B
WU6 (nM)
0
40
200
1000
Q5 33.2
Q6 24.9
Q8 19.3
Q7 22.6
Q5 30.2
Q6 13.9
Q8 31.1
Q7 24.7
Q5 26.1
Q6 8.19
Q8 34.9
Q7 30.8
Q5 14.5
Q6 8.56
Q8 37.3
Q7 39.6
CXCR3
CD8
C
WU6 (nM)
0
40
200
1000
Q17 43.0
Q18 51.2
Q20 4.32
Q19 1.50
Q17 37.3
Q18 52.1
Q20 8.01
Q19 2.64
Q17 32.5
Q18 39.9
Q20 16.9
Q19 10.7
Q17 10.6
Q18 25.5
Q20 47.1
Q19 16.8
T-bet
CD4
D
WU6 (nM)
0
40
200
1000
Q2 53.7
Q3 40.5
Q5 2.73
Q4 3.06
Q2 53.7
Q3 40.5
Q5 2.73
Q4 3.06
Q2 42.4
Q3 29.8
Q5 10.9
Q4 16.9
Q2 30.6
Q3 9.05
Q5 17.8
Q4 42.6
T-bet
CD8

Supplementary Figure S2. Effect of WU6 on the expression of CXCR3 and T-bet in vitro. Primary T cells were isolated from B6 and treated with WU6 at indicated doses for 3 days in the presence of anti-CD3/CD28 activation beads. After 3 days, the expression of CXCR3 (A-B) and T-bet (C-D) in the CD4 and CD8 was determined by flow cytometry and the representative plots were represented in A-D.
